# Shared and distinct anatomical correlates of semantic and phonemic fluency revealed by lesion-symptom mapping in patients with ischemic stroke

**DOI:** 10.1007/s00429-015-1033-8

**Published:** 2015-05-05

**Authors:** J. Matthijs Biesbroek, Martine J. E. van Zandvoort, L. Jaap Kappelle, Birgitta K. Velthuis, Geert Jan Biessels, Albert Postma

**Affiliations:** 1 Department of Neurology, Utrecht Stroke Center, Brain Center Rudolf Magnus, University Medical Center Utrecht, PO box 85500, G.03.232, 3508 GA Utrecht, The Netherlands; 2 Experimental Psychology, Helmholtz Institute, Utrecht University, Utrecht, The Netherlands; 3 Department of Radiology, University Medical Center Utrecht, Utrecht, The Netherlands

**Keywords:** Phonemic, Semantic, Fluency, Lesion-symptom mapping, Anatomical correlates, Neural substrate

## Abstract

Semantic and phonemic fluency tasks are frequently used to test executive functioning, speed and attention, and access to the mental lexicon. In semantic fluency tasks, subjects are required to generate words belonging to a category (e.g., animals) within a limited time window, whereas in phonemic fluency tasks subjects have to generate words starting with a given letter. Anatomical correlates of semantic and phonemic fluency are currently assumed to overlap in left frontal structures, reflecting shared executive processes, and to be distinct in left temporal and right frontal structures, reflecting involvement of distinct memory processes and search strategies. Definite evidence for this assumption is lacking. To further establish the anatomical correlates of semantic and phonemic fluency, we applied assumption-free voxel-based and region-of-interest-based lesion-symptom mapping in 93 patients with ischemic stroke. Fluency was assessed by asking patients to name animals (semantic), and words starting with the letter N and A (phonemic). Our findings indicate that anatomical correlates of semantic and phonemic fluency overlap in the left inferior frontal gyrus and insula, reflecting shared underlying cognitive processes. Phonemic fluency additionally draws on the left rolandic operculum, which might reflect a search through phonological memory, and the middle frontal gyrus. Semantic fluency additionally draws on left medial temporal regions, probably reflecting a search through semantic memory, and the right inferior frontal gyrus, which might reflect the application of a visuospatial mental imagery strategy in semantic fluency. These findings establish shared and distinct anatomical correlates of semantic and phonemic fluency.

**Electronic supplementary material:**

The online version of this article (doi:10.1007/s00429-015-1033-8) contains supplementary material, which is available to authorized users.

## Introduction

Verbal fluency tasks, in which participants have to generate as many words as possible according to a specific criterion, are frequently used to test a circumscribed aspect of executive functioning that is referred to as energization (i.e., the voluntary generation of non-overlearned responses) (Robinson et al. 2012). Verbal fluency additionally depends on self-monitoring (which is also regarded as an executive process), processing speed and attention (van der Elst et al. 2005), working memory (Baldo et al. 2006; Robinson et al. 2012), and language processing including retrieval of appropriate responses from the mental lexicon (Juhasz et al. 2012). Verbal fluency is typically divided into two categories: (1) semantic fluency and (2) phonemic fluency (Lezak et al. 2004). In semantic fluency tests, participants are required to generate as many items belonging to a certain category (e.g., items in a grocery store, or animals in a zoo) as possible within a certain time window. In phonemic fluency tests, the subject is asked to generate as many words starting with a given letter as possible. Though both semantic and phonemic fluency tests assess energization and self-monitoring, they require different strategies for the creation and selection of appropriate novel responses, and depend on distinct memory processes (Baldo et al. 2006). Semantic fluency draws on semantic memory to retrieve previously obtained semantic knowledge on items belonging to a certain category, whereas in phonemic fluency appropriate items are selected based on phonological word knowledge (Baldo et al. 2006). Thus, semantic and phonemic fluency depend on partially shared (energization, self-monitoring, attention, processing speed, language) and partially distinct (search strategy, semantic versus phonological memory) cognitive processes.

Delineating the anatomical correlates of semantic and phonemic fluency by patient lesion-mapping techniques would improve our understanding of the overlap and differences in the cognitive processes involved and provide valuable insight in how and why certain neurological conditions hamper semantic or phonemic fluency in individual patients. There is substantial evidence from lesion studies for a crucial role of the left frontal lobe in both semantic and phonemic fluency (Robinson et al. 2012). Regarding the specific role of left frontal structures in semantic and phonemic fluency, fMRI studies in healthy subjects have consistently demonstrated distinct dorsal–ventral locations within the left inferior frontal gyrus for semantic and phonologic processes (Costafreda et al. 2006; Heim et al. 2008, 2009; Katzev et al. 2013). The role of the right frontal lobe in verbal fluency remains controversial: some studies reported impaired verbal fluency in a proportion of patients with right frontal lesions (Perret 1974; Martin et al. 1990; Loring et al. 1994; Robinson et al. 2012), but this is not confirmed by others (Milner 1964; Newcombe 1969). It is unclear whether the potential contribution of the right frontal lobe to verbal fluency is specific for either semantic or phonemic fluency, though findings of a recent lesion study suggest that right lateral frontal regions might be involved in semantic, but not phonemic fluency (Robinson et al. 2012). Moreover, a specific role of the left temporal lobe in verbal fluency has been demonstrated. Evidence from behavioral studies in healthy participants (Martin et al. 1994), functional imaging studies (Mummery et al. 1996; Gourovitch et al. 2000), and lesion studies (Henry and Crawford 2004; Baldo et al. 2006; Robinson et al. 2012) indicates that the temporal cortex underlies semantically based word retrieval but not phonemically driven word retrieval. Though the afore mentioned studies have provided important insights into the anatomical correlates of semantic and phonemic fluency, they have not resolved the issue entirely since (1) fMRI studies in healthy subjects do not prove that activated structures are actually essential to the task, meaning that these findings should be confirmed with lesion studies to demonstrate a direct causal relation (Rorden and Karnath 2004), and (2) previously performed lesion studies have been limited by low spatial resolution or a strictly hypothesis-driven approach, thus ignoring potentially relevant brain regions. For example, the largest lesion-symptom study to date (67 patients) compared frontal lesions with posterior lesions, and additionally performed an analysis in which the frontal lobes were divided into three regions of interest (Robinson et al. 2012). Posterior lesions were associated with poor semantic fluency, but not phonemic fluency. However, due to the low spatial resolution (i.e., comparing frontal versus posterior), it remained unclear which posterior regions were crucially involved in semantic fluency. Another lesion-symptom mapping study performed voxel-wise analyses in 48 patients with left hemispheric stroke (Baldo et al. 2006). Left frontal lesions correlated with phonemic fluency, while left temporal lesions correlated with semantic fluency. A limitation of this study is that the impact of right hemispheric lesions on verbal fluency was not assessed (Baldo et al. 2006). The same limitation applies to a recent study of 31 patients with left hemispheric lesion in which an association was found between lesion in the left inferior fronto-occipital fasciculus and poor semantic, but not phonemic fluency (Almairac et al. 2014). In summary, there is a critical need for large-scale assumption-free patient lesion studies to further substantiate foregoing notions as well as resolve lingering controversies.

In the present study, we set out to further clarify the anatomical correlates of semantic and phonemic verbal fluency by applying hypothesis-free voxel-based (i.e., high spatial resolution) lesion-symptom mapping in a cohort of 93 patients with first-ever ischemic stroke. We expected that the anatomical correlates of semantic and phonemic fluency would overlap in left frontal regions and be discordant in left temporal and right frontal regions.

## Materials and methods

### Subjects

A flowchart of the inclusion of patients for the current study is provided in supplementary Fig. 1. Neuropsychological examination was performed in ischemic stroke patients who are admitted to our service in the setting of standard clinical care, if their condition permitted testing and testing facilities were available. All 243 ischemic stroke patients who were admitted from November 2005 through December 2012 and underwent neuropsychological assessment during admission were eligible for the present study (see supplementary Fig. 1). We subsequently applied a stepwise exclusion procedure to select patients without interfering pre-existent neurological conditions or brain lesions, in whom the ischemic lesion could be segmented on CT or MRI, and with available data on semantic and phonemic fluency (see supplementary Fig. 1). In the first step, we excluded 79 patients with pre-existent neurological conditions or imaging abnormalities: 19 patients with (probable) pre-existent cognitive impairment, 21 patients with prior stroke, 37 patients with old (silent) infarcts or severe white matter hyperintensity on brain imaging defined as Fazekas grade 3 [i.e., large confluent areas of white matter lesions (Fazekas et al. 1987)] on brain imaging, and 2 patients with recurrent stroke between brain imaging and neuropsychological examination. Cortical atrophy was not an exclusion criterion. In the second step, we excluded 43 patients for whom no brain imaging was available (no follow-up imaging after the acute admission scan in 24 patients, no ischemic lesion detected on follow-up imaging in 19 patients). In the final step, we excluded 28 patients who had no data on semantic and phonemic fluency. The application of these exclusion criteria resulted in the inclusion of 93 patients.

### Neuropsychological assessment

Neuropsychological assessment was performed within one month after ischemic stroke (mean 7.5 days; range 1–30 days). We have previously demonstrated that the applied cognitive assessment battery is feasible and reliable in the acute stage (first days to weeks) of ischemic stroke (Nys et al. 2005b). Measures of fluency were obtained by asking patients to name as many words as possible (in the Dutch language) in the following categories: (1) animals, in 2 min; (2) any word beginning with the letter N, in 1 min; (3) any word beginning with the letter A, in 1 min. Educational level was divided into seven categories (scored according to Verhage 1964) with scores ranging from unfinished primary school education (category 1) to an academic degree (category 7) according to the Dutch educational system.

To investigate the relationship between semantic and phonemic fluency and measures of verbal and visuospatial memory and language, we additionally considered data on the Dutch version of the Rey Auditory Verbal Learning Test (RAVLT) (Rey 1958; Brand and Jolles 1985; van der Elst et al. 2005), the delayed Rey–Osterrieth Complex Figure Test (ROCF) (Osterrieth 1944), the Boston Naming Test (short form: 30 items) (Kaplan et al. 1983), and the Token Test (short form: 21 items) (De Renzi and Vignolo 1962). These tests were administered in the same session as the fluency tests. A detailed description of the administration of the RAVLT and ROCF is provided in the online supplementary methods.

### Generation of lesion maps

The procedure for the generation of lesion maps has been previously described elsewhere (Biesbroek et al. 2014). Infarcts were manually segmented on either follow-up CT (*n* = 61), or MRI scans (*n* = 32). The infarct maps were registered to the T1 MNI-152 (Montreal Neurological Institute) template utilizing a lesion-masking approach (Brett et al. 2001; Fonov et al. 2009). Registration of MRI images was performed using elastix; CT images were registered using an in-house developed algorithm which is described elsewhere (Klein et al. 2010; Kuijf et al. 2013). A detailed description of the generation, registration and quality checks of the lesion maps is provided in the online supplementary methods.

### Statistics

Phonemic fluency was defined as the sum of correct, non-repeated words that an individual produced in the N and A letter trials. Semantic fluency was defined as the total number of correct, non-repeated animals that an individual named in the animal naming trial. Measures of phonemic and semantic fluency, and performance on the RAVLT, delayed ROCF copy test, Boston Naming Test, and Token Test were transformed to *z* scores and corrected for age, sex and level of education for each individual patient using linear regression (i.e., based on the group means and standard deviations). Pearson correlations were used to compute the correlation between semantic and phonemic fluency and measures of verbal and spatial memory. To assess the prevalence of aphasia in our study cohort, performance on the Boston Naming Test and the Token Test was dichotomized using previously described norms; performance below the 5th percentile was considered abnormal (Heesbeen 2002).

Rather than focusing on specific brain regions, patients with lesions anywhere in the brain were included. We first performed assumption-free VLSM to assess the association between the presence of a lesion and semantic and phonemic fluency in a given voxel (Rorden and Karnath 2004; Kimberg et al. 2007; Rorden et al. 2007), and complemented these voxel-wise analyses with a region-of-interest-based approach. VLSM analyses were done using Non-Parametric Mapping (most recent version, December 2012; settings: *t* test, univariate analysis) (Rorden et al. 2007). The Non-Parametric Mapping software provides two tests for VLSM: the parametric *t* test and the non-parametric Brunner–Munzel (BM) statistic. Because the *t* test has higher power than the BM statistic in small sample sizes, and because the *t* test is particularly robust as it becomes conservative rather than liberal (i.e., reporting false alarms) when the underlying assumptions are violated, we chose to use the *t* test in our main analyses (Rorden et al. 2007). Voxels affected by ischemic lesions in less than 3 patients were not considered for analysis. Correction for multiple testing was performed using a false discovery rate threshold (FDR) with *q* < 0.05. To assess the robustness of the VLSM results, we additionally performed a qualitative lesion subtraction analysis using dichotomized measures of fluency as the dependent variable (i.e., abnormal versus normal), instead of using *z* scores. Phonemic and semantic fluency were dichotomized using previously described norms that were obtained in a cohort of healthy Dutch individuals; performance below the 5th percentile was considered abnormal (Deelman et al. 1981; Brand et al. 2007; Nys et al. 2005a). Because dichotomization of performance results in a decrease in statistical power and does not account for severity of the deficit, we chose to use the continuous outcome (analyzed with *t* test) in our main analyses.

In the next step, we complemented the voxel-based analyses with a region-of-interest-based analysis to quantify the impact of regional lesion volumes on phonemic and semantic fluency. The regions of interest were selected based on the VLSM results. For this purpose, regions of interest for 90 cerebral cortical regions were extracted from the automatic anatomical labeling (AAL) atlas (Tzourio-Mazoyer et al. 2002). These 90 regions were projected on the VLSM results and the amount of voxels with a statistically significant association within each region was assessed quantitatively. Regions that appeared to be involved in phonemic or semantic fluency (operationally defined as at least 100 significant voxels within a specific region) were selected for the region-of-interest-based analyses visually. Next, infarct volume within the selected regions was calculated for every patient. These regional infarct volumes were entered as independent variables in a linear regression model with phonemic and semantic fluency as the dependent variables, before and after adding total infarct volume to the model. The rationale behind adding infarct volume as a covariate was that brain regions that are crucial when performing a certain task should predict performance, independent of total infarct volume. However, it should be kept in mind that adding infarct volume as a covariate will decrease statistical power, especially when relevant anatomical structures correlate with large infarcts (due to the anatomy of the cerebral arteries) (Karnath et al. 2004). For this reason, VLSM analyses are often not corrected for total infarct volume (Baldo et al. 2006; Haramati et al. 2008; Molenberghs and Sale 2011; Vossel et al. 2011; Fridriksson et al. 2013; Magnusdottir et al. 2013). Lesion studies in which a correction for total infarct volume is applied generally show VLSM results that are not corrected for multiple testing to compensate for reduced statistical power (Karnath et al. 2004; Schwartz et al. 2009). Instead, we chose to apply the correction for total infarct volume in the region-of-interest-based analyses because these analyses do not require correction for multiple testing (similar to Thothathiri et al. 2012; Biesbroek et al. 2014).

## Results

Clinical characteristics of the study cohort are provided in Table 1. Eighteen out of 93 patients had impaired semantic fluency (19 %); 29 (31 %) patients had impaired phonemic fluency. Thirteen patients had both impaired semantic and phonemic fluency. Impaired semantic and phonemic fluency was most prevalent in patients with left hemispheric lesions, but was also present in a substantial number of patients with right hemispheric lesions (Table 2). Semantic and phonemic fluency were significantly correlated (*r* = 0.642; *p* < 0.001). Both semantic and phonemic fluency were correlated with measures of verbal memory (working memory, delayed recall, recognition memory) and language (Boston Naming Test, Token Test) (Table 3). In contrast, semantic fluency was correlated with visuospatial memory performance (*r* = 0.233; *p* = 0.032), whereas phonemic fluency was not (*r* = 0.084; *p* = 0.444). Thirteen out of 85 patients with data on the Token Test (15 %) and 34 out of 91 patients with data on the Boston Naming Test (37 %) had impaired performance on these language tests.

**Table 1:** Characteristics of the study cohort

Characteristics	Study cohort (*n* = 93)
Demographic characteristics	
Age, mean (SD)	59.5 (14.9)
Male, *n* (%)	53 (57)
Education, median (range)^a^	5 (2–7)
Hand preference, *n* (%)^b^	
Right	82 (89)
Left	9 (10)
Ambidexter	1 (1)
Neuropsychological examination	
Time interval between stroke and NPE in days, mean (SD; range)	7.5 (5.1; 1–30)
No. words letter A (1 min)	8.0 (4.3; 0–22)
No. words letter N (1 min)	7.7 (4.4; 0–20)
No. animals (2 min)	23.1 (10.4; 0–51)

*NPE* neuropsychological examination

^a^Education scored according to Verhage scoring system (scale 1–7)

^b^Data on hand preference missing in one patient

**Table 2:** Location of ischemic lesion in relation to the presence of impaired semantic or phonemic fluency

Lesion location, *n* (%)	Impaired semantic fluency		Impaired phonemic fluency	
	Yes (*n* = 18)	No (*n* = 75)	Yes (*n* = 29)	No (*n* = 64)
Left hemisphere (*n* = 34)	11 (61 %)	23 (31 %)	16 (55 %)	18 (28 %)
Right hemisphere (*n* = 40)	5 (28 %)	35 (47 %)	8 (28 %)	32 (50 %)
Infratentorial (*n* = 12)	1 (6 %)	11 (15 %)	2 (7 %)	10 (16 %)
Multiple locations (*n* = 7)	1 (6 %)	6 (8 %)	3 (10 %)	4 (6 %)

**Table 3:** Pearson correlations between semantic and phonemic fluency and measures of language and verbal and spatial memory

	Phonemic fluency (N + A)	Semantic fluency (animal)
Semantic fluency	0.642 (*p* < 0.001)	–
RAVLT total recall trial 1–5^a^	0.558 (*p* < 0.001)	0.583 (*p* < 0.001)
RAVLT recollection^a^	0.493 (*p* < 0.001)	0.517 (*p* < 0.001)
RAVLT recognition^a^	0.425 (*p* < 0.001)	0.566 (*p* < 0.001)
Delayed ROCF^b^	0.084 (*p* = 0.444)	0.233 (*p* = 0.032)
Boston Naming Test^c^	0.405 (*p* < 0.001)	0.571 (*p* < 0.001)
Token Test^d^	0.557 (*p* < 0.001)	0.582 (*p* < 0.001)

The presented *p* values correspond with a two-tailed test

*RAVLT* Rey Auditory Verbal Learning Test, *ROCF* Rey–Osterrieth Complex Figure Test

^a^Based on 89 patients with data on the RAVLT

^b^Based on 85 patients with data on delayed ROCF

^c^Based on 91 patients with data on Boston Naming Test

^d^Based on 85 patients with data on Token Test

### Voxel-based lesion-symptom mapping

**Fig. 1:**
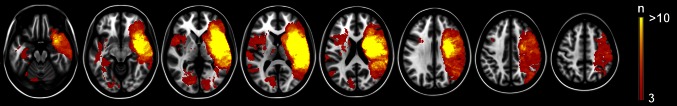
Distribution of ischemic lesions. Voxels that are damaged in at least three patients are projected on the 1 mm MNI-152 template (*Z* coordinates: −20, −10, 0, 10, 20, 30, 40, 50). *Bar* the number of patients with a lesion for each voxel. The right hemisphere is depicted on the *right*

**Fig. 2:**
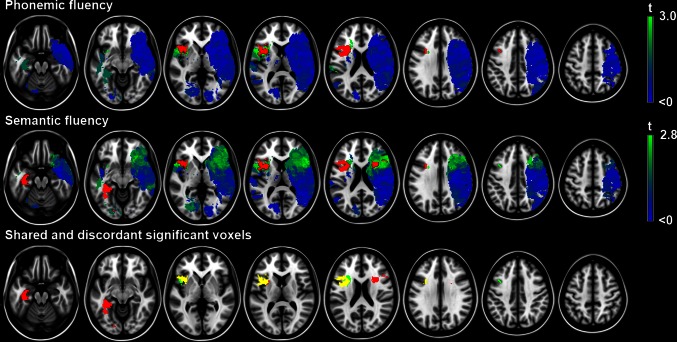
Voxel-based lesion-symptom mapping results. Map of the voxel-wise association (*t* statistic) between the presence of a lesion and cognitive performance. Voxels exceeding the false discovery rate threshold (*q* = 0.05) are rendered in *red*. Non-significant voxels are rendered on a scale from *blue* (*t* < 0) to *bright green* (*t* value just below threshold). Negative *t* values (meaning the presence of a lesion was correlated with better cognitive performance) were not statistically significant. *Lower row* voxels with a statistically significant inverse association with performance on either semantic fluency (*red*), phonemic fluency (*green*) or both (*yellow*) are depicted. Note that the anatomical correlates overlap in left frontal regions, but are discordant in left temporal and right frontal regions. Semantic and phonemic fluency were corrected for age, sex and level of education using linear regression. The results are projected on the MNI 1-mm template (*Z* coordinates: −20, −10, 0, 10, 20, 30, 40, 50). The right hemisphere is depicted on the *right*

**Fig. 3:**
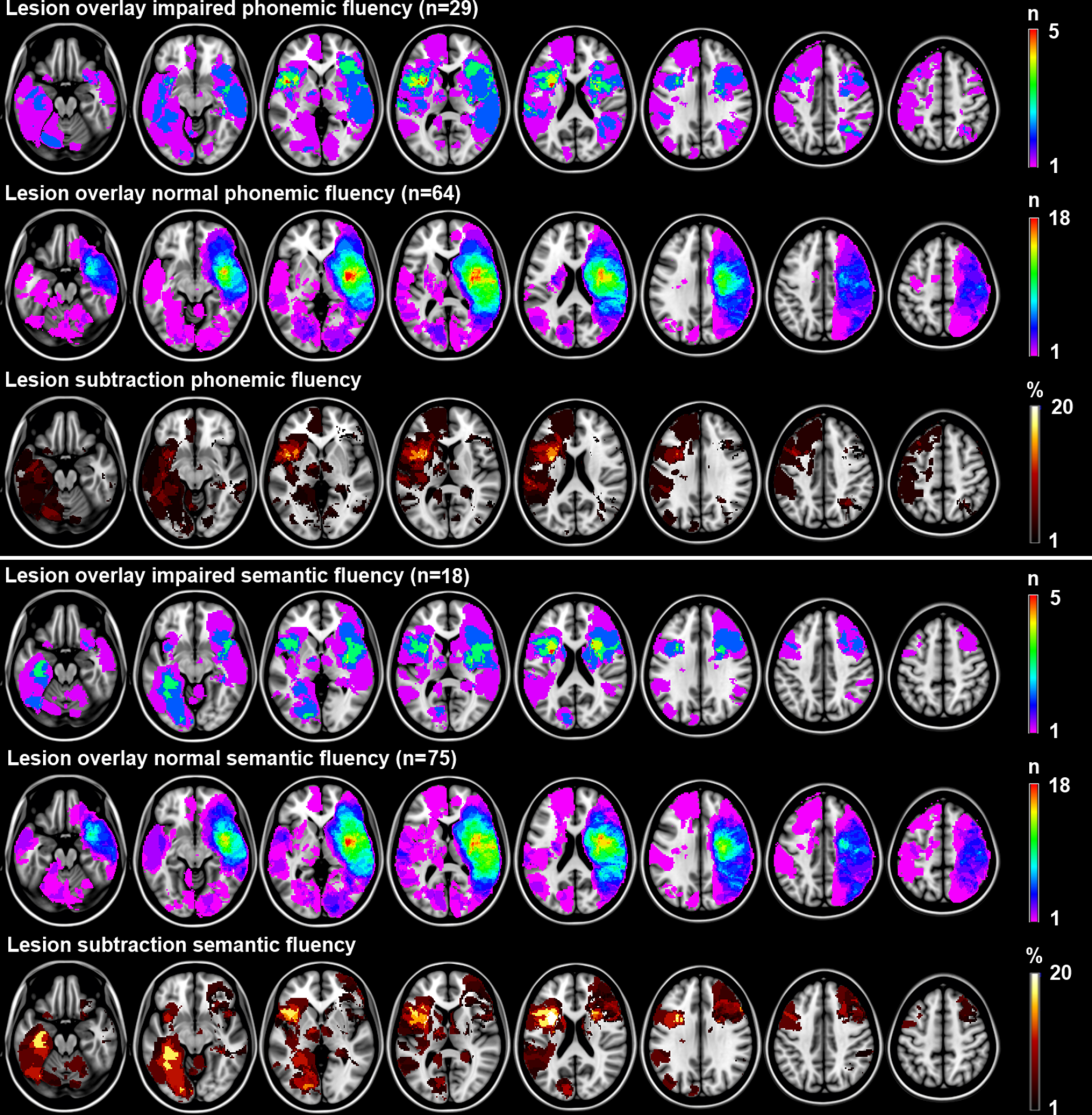
Lesion subtraction analyses with dichotomized fluency measures as outcome. Lesion overlay and subtraction plots of dichotomized measures of fluency (impaired yes/no based on previously described norms). The *overlay plots* show the number of patients with a lesion for a given voxel separately for patients with impaired and normal performance. The *lesion subtraction plots* show which voxels are more frequently affected in patients with impaired performance compared to patients with normal performance. For example, the semantic fluency overlay plots show that 3 out of 18 patients (17 %) with impaired semantic fluency have a lesion in the left hippocampus, whereas none of the 75 (0 %) patients with normal semantic fluency have a lesion in the left hippocampus. The lesion subtraction plot shows the resulting 17 % difference in lesion prevalence. This finding suggests a crucial role of the left hippocampus in semantic fluency. The lesion subtraction and voxel-based lesion-symptom mapping results are essentially the same: phonemic and semantic fluency both depend on left frontal structures. Semantic fluency additionally depends on left medial temporal and right frontal structures, whereas phonemic fluency does not. The right hemisphere is depicted on the *right*

The spatial distribution of infarcts is illustrated by the lesion prevalence map in Fig. 1. Lesion prevalence was highest for voxels in the right cerebral hemisphere in the vascular territory of the middle cerebral artery. VLSM identified large overlapping anatomical correlates for semantic and phonemic fluency in the left frontal lobe (inferior and medial frontal, and precentral gyri, and rolandic operculum, insula, and putamen). Anatomical correlates were discordant in the following regions: lesions in the left medial temporal lobe (hippocampus, and perihippocampal, inferior temporal, lingual, and fusiform gyri) and right frontal lobe (inferior frontal gyrus and periventricular white matter) were associated with poor semantic, but not phonemic fluency. In contrast, lesions in the left middle frontal gyrus were associated with poor phonemic, but not semantic fluency. The VLSM results for semantic and phonemic fluency are provided in Fig. 2. The number of significant voxels for each region is provided in Table 4. To assess the robustness of the VLSM results, we additionally performed a qualitative lesion subtraction analysis using dichotomized cognitive performance as the dependent variable, instead of using *z* scores. The results of these lesion subtraction analyses were essentially the same as the VLSM results (Fig. 3).

**Table 4:** Voxel-based lesion-symptom mapping results: tested and significant voxels for each region of interest

Anatomical regions (AAL atlas)	Patients with lesion (*n*)*	Region size, voxels (*n*)	Tested voxels (*n*)	Significant voxels semantic [*n* (%)]	Significant voxels phonemic [*n* (%)]
Middle frontal gyrus L	7	38,722	111	7 (6.3)	111 (100)
Inferior frontal gyrus operc L	8	8271	2926	1776 (60.7)	1832 (62.6)
Inferior frontal gyrus triang L	8	20,104	593	559 (94.3)	581 (98.0)
Rolandic operculum L	11	7939	2670	682 (25.5)	717 (26.9)
Insula L	17	15,025	5314	3158 (59.4)	3485 (65.6
Precentral gyrus L	13	28,174	766	340 (44.4)	436 (56.9)
Putamen L	17	7942	1695	383 (22.6)	297 (17.5)
Hippocampus L	8	7469	874	774 (88.6)	0
Parahippocampal gyrus L	4	7891	843	839 (99.5)	0
Fusiform gyrus L	8	18,333	3874	2615 (67.5)	0
Inferior temporal gyrus L	7	25,647	550	329 (59.8)	0
Lingual gyrus L	12	16,932	4543	156 (3.4)	0
Inferior frontal gyrus operc R	27	11,174	9340	107 (1.1)	0
Inferior frontal gyrus triang R	20	17,132	11,470	158 (1.4)	0

Regions that appeared to be involved in semantic or phonemic fluency are shown (definition: significant association between lesion and performance in at least 100 voxels). The remaining 76 regions contained <100 significant voxels for both semantic and phonemic fluency; these regions are not shown here

*R* right, *L* left

* How many of the 93 included patients had a lesion that overlapped (≥1 voxel) with the specified region of interest

### Region-of-interest-based analyses

Next, we analyzed the impact of lesion volumes within specific cortical regions of interest on semantic and phonemic fluency (Table 5). Infarct volume within the left inferior frontal gyrus and left insula inversely correlated with both semantic and phonemic fluency. Infarct volume within the left rolandic operculum and left medial frontal gyrus inversely correlated with phonemic, but not semantic fluency; in contrast, infarct volume within the left putamen inversely correlated with semantic, but not phonemic fluency. The discordance of anatomical correlates in the left medial temporal lobe and right frontal lobe was reproduced: there was a statistically significant inverse correlation between infarct volume within the left hippocampus, perihippocampal, inferior temporal, lingual, and fusiform gyri, and the right inferior frontal gyrus, and semantic fluency, but not phonemic fluency. The impact of regional infarct volume on semantic fluency was greatest in the left parahippocampal gyrus (unstandardized coefficient (*B*) −0.45; 95 % CI −0.75 to −0.15); the impact of regional infarct volume on phonemic fluency was greatest in the left rolandic operculum (*B* −0.44; 95 % CI −0.70 to −0.17)). The results of the linear regression analyses remained essentially the same after additional adjustment for total infarct volume (supplementary Table 1).

**Table 5:** Results of linear regression models with *z* scores of cognitive performance as outcome

Model	Independent variables	Semantic fluency			Phonemic fluency		
		*R* ^2^	*p*∆*R* ^2^	*B* (95 % CI)	*R* ^2^	*p*∆*R* ^2^	*B* (95 % CI)
1	Age, sex, level of education	0.057	0.155		0.095	0.031	
Left frontal regions							
2a	Model 1 + IV L middle frontal gyrus	0.064	0.424	−0.05 (−0.19 to 0.08)	0.155	0.014	−0.15 (−0.27 to −0.03)
2b	Model 1 + IV L inferior frontal gyrus opercular part	0.163	0.001	−0.35 (−0.56 to −0.14)	0.200	0.001	−0.33 (−0.52 to −0.14)
2c	Model 1 + IV L inferior frontal gyrus triangular part	0.142	0.004	−0.37 (−0.62 to −0.12)	0.181	0.003	−0.35 (−0.58 to −0.12)
2d	Model 1 + IV L rolandic operculum	0.066	0.360	−0.14 (−0.44 to 0.16)	0.192	0.002	−0.44 (−0.70 to −0.17)
2e	Model 1 + IV L insula	0.106	0.031	−0.16 (−0.31 to −0.02)	0.230	<0.001	−0.26 (−0.39 to −0.13)
2f	Model 1 + IV L precentral gyrus	0.065	0.388	−0.08 (−0.26 to 0.10)	0.110	0.215	−0.10 (−0.27 to 0.06)
2g	Model 1 + IV L putamen	0.103	0.035	−0.41 (−0.80 to −0.03)	0.120	0.115	−0.29 (−0.65 to 0.07)
Right frontal regions							
2h	Model 1 + IV R inferior frontal gyrus opercular part	0.130	0.008	−0.13 (−0.22 to −0.04)	0.096	0.709	−0.02 (−0.11 to 0.07)
2i	Model 1 + IV R inferior frontal gyrus triangular part	0.128	0.009	−0.11 (−0.19 to −0.03)	0.096	0.730	−0.01 (−0.09 to 0.07)
Left temporal regions							
2j	Model 1 + IV L hippocampus	0.144	0.004	−0.45 (−0.74 to −0.15)	0.119	0.123	−0.22 (−0.51 to 0.06)
2k	Model 1 + IV L parahippocampal gyrus	0.145	0.003	−0.45 (−0.75 to −0.15)	0.121	0.110	−0.23 (−0.52 to 0.05)
2l	Model 1 + IV L fusiform gyrus	0.136	0.006	−0.15 (−0.26 to −0.05)	0.115	0.162	−0.07 (−0.17 to 0.03)
2m	Model 1 + IV L Inferior temporal gyrus	0.133	0.006	−0.16 (−0.27 to −0.04)	0.120	0.111	−0.09 (−0.19 to 0.02)
2n	Model 1 + IV L Lingual gyrus L	0.106	0.030	−0.13 (−0.26 to −0.01)	0.108	0.249	−0.07 (−0.18 to 0.05)

The explained variance (*R* ^2^) in semantic and phonemic fluency is given for each model with the corresponding *p* value for the difference in explained variance (∆*R* ^2^) between the model and the previous model. Unstandardized coefficients (*B*) with corresponding 95 % CIs are provided. The unstandardized coefficient applies to the change in *z* score for every 1 ml increase in infarct volume

*IV* infarct volume, *L* left, *R* right

## Discussion

The findings of the current study indicate that semantic and phonemic fluency have partially shared and partially distinct neural underpinnings. Anatomical correlates overlap in the left inferior frontal gyrus and insula, reflecting shared underlying cognitive processes. Phonemic fluency additionally draws on the left rolandic operculum and the left middle frontal gyrus. In contrast, left medial temporal regions and the right inferior frontal gyrus are crucially involved in semantic, but not phonemic fluency.

The main strengths of the current study are the substantial sample size, the assumption-free nature of the analyses (as opposed to hypothesis-driven analyses, in which the analyses are focused on predefined regions of interest), and the application of quantitative voxel-wise analyses that provides good spatial resolution. Our findings regarding the crucial role of left frontal structures in both semantic and phonemic fluency and involvement of the left temporal lobe in semantic, but not phonemic fluency are in line with previous findings (Martin et al. 1994; Mummery et al. 1996; Gourovitch et al. 2000; Henry and Crawford 2004; Baldo et al. 2006; Robinson et al. 2012). However, these previous studies focused on the left hemisphere, or compared posterior/temporal lesions in either hemisphere with frontal regions. As such, the current study is the first to determine the anatomical correlates of semantic and phonemic fluency in an assumption-free, voxel-wise manner, taking into account lesions in both hemispheres. Furthermore, our findings provide new insights in the involvement of the right frontal lobe in verbal fluency: lesions in the right inferior frontal gyrus and periventricular frontal white matter are associated with poor semantic, but not phonemic fluency. Thus, right dorsolateral frontal structures are involved in semantic, but not phonemic fluency.

The observed partially shared and partially discordant anatomical correlates of semantic fluency reflect the involvement of multi-component cognitive processes. The shared correlates in the left inferior frontal gyrus and insula are likely to reflect word production and processing. This is further underlined by the strong correlations between both semantic and phonemic fluency, and the Boston Naming Test and Token Test. The observed involvement of the left rolandic operculum in phonemic fluency, but not in semantic fluency might reflect a search through phonological memory. In contrast, involvement of left medial structures in semantic fluency might reflect a search through semantic memory. Indeed, a crucial role of left medial temporal structures in verbal semantic memory has been clearly established (Tulving and Markowitsch 1998; Levy et al. 2004; Binder et al. 2009; Groussard et al. 2010), while perisylvian regions (including the rolandic operculum) are known to be involved in accessing phonological representations and phoneme selection and production (Alexander and Hillis 2008).

To our knowledge, there is currently no well-established theory that would explain why right dorsolateral frontal structures are involved in semantic, but not phonemic fluency. We speculate here that the differential involvement of right frontal regions might reflect a ‘visuospatial mental imagery strategy’, in which the subject generates mental images of appropriate items (animals in our case). Such a strategy could be helpful when searching through semantic memory, but would not be appropriate when searching through phonological memory. The application of a strategy involving mental imagery of concrete things in semantic fluency tasks has been previously suggested, based on the observation that patients often report imagining themselves walking through a zoo or a farm when asked to name as many animals as possible (Baldo et al. 2006). The right dorsolateral prefrontal cortex is known to be involved in keeping spatial information ‘on-line’ and in strategy formation (Miotto et al. 1996; van Asselen et al. 2006). Thus, the application of a visuospatial mental imagery strategy in semantic fluency would likely depend on right dorsolateral frontal regions. Furthermore, the observed correlation of performance on the visuospatial memory test with semantic fluency, but not with phonemic fluency (Table 3), would fit with the application of a visuospatial mental imagery strategy in semantic fluency. The process of mental imagery of animals in semantic fluency tasks can perhaps be compared to the imagery that is needed for design fluency tasks, which would fit with previous findings that right lateral frontal lesions result in impaired design fluency (Robinson et al. 2012). Unfortunately, we have no data on (visuospatial) strategy formation to further substantiate this hypothesis. Further studies are needed to determine whether the contribution of right dorsolateral frontal structures indeed reflects the application of a visuospatial mental imagery strategy in semantic fluency.

A potential limitation of the current study is the relatively low lesion frequency in the left cerebral hemisphere in the voxel-based analyses (despite the substantial number of patients with left hemispheric lesions). The reason for this lies in the fact that neuropsychological examination is not always feasible in patients with severe global aphasia, especially when applying tests that require processing of verbal information. The decision whether or not to perform a neuropsychological examination in patients with global aphasia was made by the treating clinical neuropsychologist as these tests were always performed in the setting of standard clinical care. The presence of aphasia was not an exclusion criterion for the current study. Despite the relatively low lesion frequency in left hemispheric voxels, we were able to demonstrate differential involvement of left temporal and left frontal regions in semantic and phonemic fluency. Second, we used both CT and MRI scans for lesion segmentation, which is not uncommon in lesion-symptom mapping studies in stroke (Karnath et al. 2004; Schwartz et al. 2009; Thothathiri et al. 2012; Robinson et al. 2012; Theys et al. 2013). Both modalities allow for accurate detection of the location on the ischemic lesion. However, the boundary of the lesion might be drawn differently between modalities. In addition, this boundary is also influenced by the elapsed time between stroke onset and CT/MRI scan acquisition. The variability in lesion segmentation could be minimized by applying a single scan modality in a certain time window (e.g., MRI acquired 48–72 h after stroke onset). However, we chose for a robust design including as many patients as possible (with either CT or MRI scans) to optimize statistical power, while accepting some heterogeneity in scan acquisition (Biesbroek et al. 2014). It should be noted that the marked differences in anatomical correlates of discriminability in the left temporal and right frontal lobe cannot be attributed to slight variability in the segmentation of lesion boundaries. The level of difficulty of fluency tests may differ per letter (for phonological fluency) and category (for semantic fluency) and depends on task duration as well (i.e., 1 versus 2 min). The level of difficulty of the test could affect the function–structure mapping because an increased level of difficulty might theoretically result in recruitment of nonspecific brain regions (Dräger et al. 2004). However, this cannot explain the observed association between lesions in right frontal and left temporal regions and poor semantic, but not phonemic fluency, because the semantic fluency test was in fact less difficult than the phonemic fluency tests. Patients on average named more animals (mean of 23 in 2 min) than words starting with the letter N and A combined (mean of 16 words in 2 min); see Table 1. Furthermore, we did not directly compare performance on both tests (which would be problematic because of the assumed differences in difficulty). Instead, performance on each test was transformed to *z* scores based on individual variation in test performance. We subsequently identified the anatomical correlates of each task separately, followed by a qualitative comparison to identify shared and unique anatomical correlates.

In conclusion, our findings indicate that both semantic and phonemic fluency depend on left frontal structures, while left medial temporal and right dorsolateral frontal structures are involved in semantic, but not phonemic fluency. The involvement of left medial temporal regions in semantic fluency most likely reflects retrieval of appropriate responses from semantic memory. Phonemic fluency depends more strongly on left perisylvian regions which might reflect retrieval of responses from phonological memory. The involvement of right dorsolateral frontal regions in semantic, but not phonemic, might reflect the application of a spatial strategy.

## Electronic supplementary material

Below is the link to the electronic supplementary material.

Supplementary material 1 (DOCX 15 kb)

Supplementary material 2 (DOCX 14 kb)

Supplementary material 3 (TIFF 61 kb). Flowchart of the inclusion of patients
