# Supplementary material for: Shared and distinct anatomical correlates of semantic and phonemic fluency revealed by lesion-symptom mapping in patients with ischemic stroke
Source: Brain Struct Funct. 2015 May 5;221(4):2123–34. doi: 10.1007/s00429-015-1033-8 (PMC4853441; doi:10.1007/s00429-015-1033-8)
Supplement: Supplementary file 1 — Supplementary material 1 (DOCX 15 kb) [file 429_2015_1033_MOESM1_ESM.docx]

**SUPPLEMENTARY METHODS**

**Neuropsychological examination**

When performing the RAVLT, patients were presented with 15 common, monosyllabic words in auditory format. Directly thereafter, they were asked to repeat as many of the presented words as possible. The trial was repeated four more times, in which the same words were presented in identical order. Following the five consecutive trials, a battery of unrelated tests was conducted for approximately 20 minutes. After the delay, participants had again to recall the words of the initial test. This was immediately followed by a delayed recognition test: the 15 words of the initial test (target words) were mixed with 15 new words (distracter words) and the participants were asked to indicate for each word whether it was a target or not (yes/no). To perform the ROCF copy test, the figure was placed in front of the subject who was requested to copy the figure as accurately as possible. After a delay of 10 minutes, the patient was asked to reproduce the figure from memory.

**Generation of lesion maps**

All scans were performed in the setting of standard clinical care. CT scans were acquired using a Philips Mx8000 16, Brilliance 64, or Brilliance 256 CT scanner. MRI scans were acquired using either a Philips Intera (1.5 Tesla) or Philips Achieva (1.5 or 3.0 Tesla) scanner. Only CT scans that were performed >48 hours after the onset of clinical symptoms were used; this criterion was not used for MRI scans because diffusion-weighted imaging (DWI) allows for detection of the infarct even within hours from symptom onset (Jauch *et al.*, 2013). Forty-nine out of 61 CT scans (80 %) were performed within 8 days of the onset of symptoms, while the remaining 12 CT scans were performed >20 days after the onset of symptoms. It is therefore unlikely that the “fogging effect” (transient reversal of CT hypodensities that sometimes occurs 2-3 weeks after ischemia because the edema resolves while the cavitation phase has not yet occurred) has influenced the segmentation results (Yannes *et al.*, 2013). Segmentations were performed by 2 trained reviewers (initial segmentation by N.A.W. (see acknowledgments section), segmentation reviewed by J.M.B., followed by consensus review if necessary) who were blinded to neuropsychological data to prevent potential bias. Segmentations of both CT and MRI scans were performed on transversal slices of 4-6 mm width using software that was developed for this purpose in MeVisLab (MeVis Medical Solutions AG, Bremen, Germany) (Ritter *et al.*, 2011). Infarct segmentation on MRI scans was performed on the T2 FLAIR sequence; in all 32 cases, the reviewers additionally had access to the diffusion-weighted imaging (DWI) and apparent diffusion coefficient (ADC) images. The DWI and ADC images served to ensure accurate segmentation of hyperacute ischemic stroke which is visible on DWI, while the full extent of the infarct might not yet be visible on the FLAIR sequence (this was the case in two patients who underwent MRI within 24 hours). The DWI and ADC images also served to discriminate between new infarcts and pre-existent white matter lesions. We have previously shown that the intra- and interobserver agreement for infarct segmentation on CT is high (Biesbroek et al, 2014, online supplement). The intra- and interobserver reliability of infarct segmentation on MRI is known to be high as well (Fiez *et al.*, 2000).

Because T1 sequences were not available for all patients, the T2 FLAIR was registered directly to the T1 MNI-152 (Montreal Neurological Institute) template (Fonov *et al.*, 2009). Registration was performed using elastix, with a linear registration followed by a non-linear registration (Klein *et al.*, 2010). For registration to standard space, a lesion-masking approach was applied to enhance registration quality; lesion masks were created by subtracting infarct lesion maps from brain masks (Brett *et al.*, 2001). After registration of the T2 FLAIR, the warp fields were used to co-register the corresponding infarct maps to the 1-mm MNI template. The same registration method was applied to the CT scans using a registration algorithm that was designed and validated for this purpose; this algorithm is described elsewhere (Kuijf *et al.*, 2013). Rigorous quality checks of the registration results were performed by comparing the native scan to the lesion map in MNI space. The co-registered lesion maps of 29 patients were manually adjusted to correct for slight registration errors using MRIcron (http://www.mccauslandcenter.sc.edu/mricro/mricron). Adjustment of the lesion maps was performed by one reviewer (J.M.B.) who was blinded to neuropsychological data. The registration, followed by manual adjustments in 29 cases, resulted in an accurate translation of the lesion maps to standard space for all 93 patients.

**SUPPLEMENTARY REFERENCES**

# - Biesbroek JM, van Zandvoort MJ, Kuijf HJ, Weaver NA, Kappelle LJ, Vos PC, et al. The anatomy of visuospatial construction revealed by lesion-symptom mapping. Neuropsychologia 2014; 62: 68-76.

- Brett M, Leff AP, Rorden C, Ashburner J. Spatial normalization of brain images with focal lesions using cost function masking. Neuroimage 2001; 14: 486–500.

- Fiez JA, Damasio H, Grabowski TJ. Lesion segmentation and manual warping to a reference brain: intra- and interobserver reliability. Hum Brain Mapp 2000; 9: 192-211.

- Fonov V, Evans A, McKinstry R, Almli C, Collins D. Unbiased nonlinear average age-appropriate brain templates from birth to adulthood. Neuroimage 2009; 47: S102.

**-** Jauch EC, Saver JL, Adams HP Jr, Bruno A, Connors JJ, Demaerschalk BM, et al: Guidelines for the early management of patients with acute ischemic stroke: a guideline for healthcare professionals from the American Heart Association/American Stroke Association. Stroke 2013; 44: 870–947

- Klein S, Staring M, Murphy K, Viergever MA, Pluim JP. Elastix: A toolbox for intensity-based medical image registration. IEEE Transactions on Medical Imaging 2010; 29: 196–205.

- Kuijf HJ, Biesbroek JM, Viergever MA, Biessels GJ, Vincken KL. Registration of brain CT images to an MRI template for the purpose of lesion-symptom mapping. Multimodal Brain Image Analysis, Lecture Notes in Computer Science 2013; 8159: 119-28.

- Ritter F, Boskamp T, Homeyer A, Laue H, Schwier M, Link F, Peitgen H-O. Medical Image Analysis: A visual approach. IEEE Pulse 2011; 2: 60–70.

- Yannes M, Frabizzio J, Shah QA. Reversal of CT hypodensity after acute ischemic stroke. J Vasc Interv Neurol 2013; 6: 10-14.
