# Supplementary material for: Shared and distinct anatomical correlates of semantic and phonemic fluency revealed by lesion-symptom mapping in patients with ischemic stroke
Source: Brain Struct Funct. 2015 May 5;221(4):2123–34. doi: 10.1007/s00429-015-1033-8 (PMC4853441; doi:10.1007/s00429-015-1033-8)
Supplement: Supplementary file 2 — Supplementary material 2 (DOCX 14 kb) [file 429_2015_1033_MOESM2_ESM.docx]

**Supplementary Table 1. Results of linear regression models with z-scores of cognitive performance as outcome after correction for total infarct volume.**

|  |  | Flu semantic | | | Flu phonemic | | |
| --- | --- | --- | --- | --- | --- | --- | --- |
| Model | Independent variables | R^2^ | p∆R^2^ | B (95% CI) | R^2^ | p ∆R^2^ | B (95% CI) |
| 1 | Age, sex, level of education | 0.057 | 0.155 |  | 0.095 | 0.031 |  |
| 2 | Model 1 + total IV | 0.105 | **0.032** | -0.01 (-0.01 to 0.00) | 0.098 | 0.577 | -0.00 (-0.01 to 0.00) |
| **Left frontal regions** | |  |  |  |  |  |  |
| 3a | Model 1 + IV L middle frontal gyrus | 0.110 | 0.480 | -0.05 (-0.18 to 0.08) | 0.157 | **0.015** | -0.15 (-0.27 to -0.03) |
| 3b | Model 1 + IV L inferior frontal gyrus opercular part | 0.214 | **0.001** | -0.35 (-0.56 to -0.15) | 0.204 | **0.001** | -0.33 (-0.52 to -0.14) |
| 3c | Model 1 + IV L inferior frontal gyrus triangular part | 0.191 | **0.003** | -0.37 (-0.61 to -0.13) | 0.184 | **0.003** | -0.35 (-0.58 to -0.12) |
| 3d | Model 1 + IV L rolandic operculum | 0.115 | 0.342 | -0.14 (-0.44 to 0.15) | 0.195 | **0.002** | -0.44 (-0.71 to -0.17) |
| 3e | Model 1 + IV L insula | 0.156 | **0.025** | -0.17 (-0.31 to -0.02) | 0.234 | **<0.001** | -0.26 (-0.39 to -0.13) |
| 3f | Model 1 + IV L precentral gyrus | 0.115 | 0.339 | -0.08 (-0.26 to 0.09) | 0.114 | 0.209 | -0.11 (-0.27 to 0.06) |
| 3g | Model 1 + IV L putamen | 0.157 | **0.023** | -0.44 (-0.81 to -0.06) | 0.124 | 0.110 | -0.30 (-0.66 to 0.07) |
| **Right frontal regions** | |  |  |  |  |  |  |
| 3h | Model 1 + IV R inferior frontal gyrus opercular part | 0.131 | 0.115 | -0.12 (-0.26 to 0.03) | 0.098 | 0.941 | 0.01 (-0.13 to 0.14) |
| 3i | Model 1 + IV R inferior frontal gyrus triangular part | 0.128 | 0.133 | -0.10 (-0.23 to 0.03) | 0.098 | 0.894 | 0.01 (-0.12 to 0.13) |
| **Left temporal regions** | |  |  |  |  |  |  |
| 3j | Model 1 + IV L hippocampus | 0.177 | **0.007** | -0.41 (-0.70 to -0.11) | 0.120 | 0.140 | -0.22 (-0.51 to 0.07) |
| 3k | Model 1 + IV L parahippocampal gyrus | 0.177 | **0.007** | -0.42 (-0.71 to -0.12) | 0.122 | 0.127 | -0.23 (-0.52 to 0.07) |
| 3l | Model 1 + IV L fusiform gyrus | 0.169 | **0.011** | -0.14 (-0.24 to -0.03) | 0.116 | 0.184 | -0.07 (-0.17 to 0.03) |
| 3m | Model 1 + IV L Inferior temporal gyrus | 0.168 | **0.012** | -0.14 (-0.25 to -0.03) | 0.122 | 0.127 | -0.08 (-0.19 to 0.02) |
| 3n | Model 1 + IV L Lingual gyrus L | 0.145 | **0.048** | -0.12 (-0.24 to -0.00) | 0.110 | 0.274 | -0.06 (-0.18 to 0.05) |

The explained variance (R^2^) in semantic and phonemic fluency is given for each model with the corresponding p-value for the difference in explained variance (∆ R^2^) between the model and the previous model. Unstandardized coefficients (B) with corresponding 95% CIs are provided. The unstandardized coefficient applies to the change in z-score for every 1 ml increase in infarct volume. IV: infarct volume. L: left. R: right.
