# Supplementary figures and images for: Shared and distinct anatomical correlates of semantic and phonemic fluency revealed by lesion-symptom mapping in patients with ischemic stroke
Source: Brain Struct Funct. 2015 May 5;221(4):2123–34. doi: 10.1007/s00429-015-1033-8 (PMC4853441; doi:10.1007/s00429-015-1033-8)

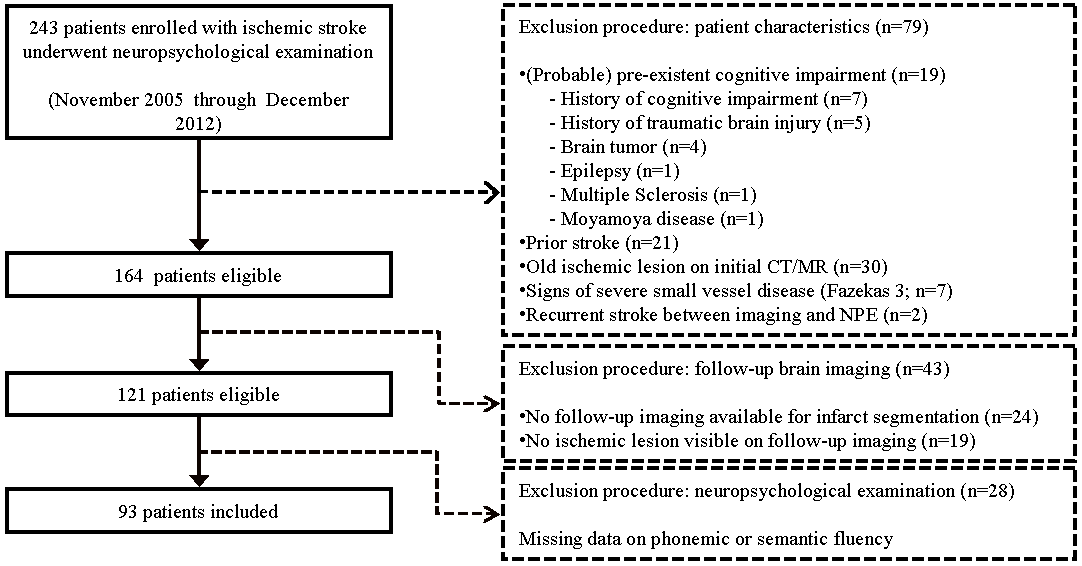

Supplement: Supplementary file 3 — Supplementary material 3 (TIFF 61 kb). Flowchart of the inclusion of patients [file 429_2015_1033_MOESM3_ESM.tif]
